# A comparative analysis of gene expression profiling by statistical and machine learning approaches

**DOI:** 10.1093/bioadv/vbae199

**Published:** 2024-12-18

**Authors:** Myriam Bontonou, Anaïs Haget, Maria Boulougouri, Benjamin Audit, Pierre Borgnat, Jean-Michel Arbona

**Affiliations:** CNRS, ENS de Lyon, Inserm, LBMC, UMR5239, U1293, F-69342 Lyon Cedex 07, France; CNRS, ENS de Lyon, LPENSL, UMR5672, F-69342 Lyon Cedex 07, France; LTS2 Laboratory, EPFL, 1015 Lausanne, Switzerland; LTS2 Laboratory, EPFL, 1015 Lausanne, Switzerland; CNRS, ENS de Lyon, LPENSL, UMR5672, F-69342 Lyon Cedex 07, France; CNRS, ENS de Lyon, LPENSL, UMR5672, F-69342 Lyon Cedex 07, France; CNRS, ENS de Lyon, Inserm, LBMC, UMR5239, U1293, F-69342 Lyon Cedex 07, France

## Abstract

**Motivation:**

Many machine learning (ML) models developed to classify phenotype from gene expression data provide interpretations for their decisions, with the aim of understanding biological processes. For many models, including neural networks, interpretations are lists of genes ranked by their importance for the predictions, with top-ranked genes likely linked to the phenotype. In this article, we discuss the limitations of such approaches using integrated gradient, an explainability method developed for neural networks, as an example.

**Results:**

Experiments are performed on RNA sequencing data from public cancer databases. A collection of ML models, including multilayer perceptrons and graph neural networks, are trained to classify samples by cancer type. Gene rankings from integrated gradients are compared to genes highlighted by statistical feature selection methods such as DESeq2 and other learning methods measuring global feature contribution. Experiments show that a small set of top-ranked genes is sufficient to achieve good classification. However, similar performance is possible with lower-ranked genes, although larger sets are required. Moreover, significant differences in top-ranked genes, especially between statistical and learning methods, prevent a comprehensive biological understanding. In conclusion, while these methods identify pathology-specific biomarkers, the completeness of gene sets selected by explainability techniques for understanding biological processes remains uncertain.

**Availability and implementation:**

Python code and datasets are available at https://github.com/mbonto/XAI_in_genomics.

## 1 Introduction

A phenotype results from a complex cascade of molecular processes possibly involving hundreds or thousands of genes. Understanding the connections between phenotype and genes is crucial to better identify mechanisms implicated in diseases such as cancer.

With the advent of genome-wide expression profiling (i.e. from microarray chips to RNA sequencing), a key question has been to which extent gene expression (gene activity) changes associated with phenotypes provide biological insights. Over the years, several machine learning (ML) models have been proposed for the diagnosis of cancers (Golub *et al.* 1999, Li *et al.* 2017, Ahn *et al.* 2018, Hoadley *et al.* 2018, Ramirez *et al.* 2020, Hanczar *et al.* 2022) and the identification of cancer subtypes (Kohavi and John 1997, Xiong *et al.* 2001, Nguyen and Rocke 2002, Krishnapuram *et al.* 2004, Parker *et al.* 2009, Leng *et al.* 2022, Rohimat *et al.* 2022, Jacquet *et al.* 2023) from genes activity in patients’tissues. Models such as naive Bayes classifiers (Kohavi and John 1997), discriminant analyses (Xiong *et al.* 2001, Nguyen and Rocke 2002), k-nearest neighbours classifiers (Li *et al.* 2017), support vector machines (Krishnapuram *et al.* 2004, Rohimat *et al.* 2022), XGBoost (Li *et al.* 2017, Hanczar *et al.* 2022), LASSO regressions (Krishnapuram *et al.* 2004, Hanczar *et al.* 2022), and more recently neural networks [mostly multilayer perceptrons (MLPs) (Ahn *et al.* 2018, Hanczar *et al.* 2022, Leng *et al.* 2022) and graph neural networks (GNNs) (Ramirez *et al.* 2020, Leng *et al.* 2022)] have been used. These recent neural networks have the advantage of being more expressive, in other words theoretically capable of learning more complex relationships linking phenotypes to gene expression (Goodfellow *et al.* 2016).

In a medical setting, the accuracy (reliability of the models) and interpretability (understanding of the factors leading a model to a decision) are crucial. Interpretable ML (Molnar 2022) and explainability techniques for neural networks offer ways to explain the decisions of the classifiers by determining discriminative genes. Many techniques attribute a score to the genes proportional to their importance for the model. These scores allow to rank genes and possibly select the most relevant ones as biomarkers to explain specific classes. In this context, gene expression profiling refers to the creation of a profile of genes ranked according to their relevance for predicting (and possibly understanding) phenotypes.

For some models, such as XGBoost and logistic regressions (LRs), interpretability techniques offer global insights into feature contribution. For black-box neural networks, which contain too many parameters for their overall decision process to be easily understood, local scores derived from *post-hoc* explainability methods (computed after training) identify the most important genes for each prediction (Mahendran *et al.* 2020, Ramirez *et al.* 2020, Alharbi and Vakanski 2023). These scores can then be aggregated into a global score per gene to better understand the model.

Gene expression datasets are high dimensional with a relatively small sample size. To improve the performance of some classifiers, discriminative genes are sometimes pre-selected before training (Kohavi and John 1997, Mahendran *et al.* 2020, Alharbi and Vakanski 2023) with feature selection methods, for instance with statistical tests (Xiong *et al.* 2001, Parker *et al.* 2009, Jacquet *et al.* 2023) or on correlation measures to remove redundant genes (Kohavi and John 1997, Yu and Liu 2004). To address data redundancy during training, models like regularized LR and XGBoost incorporate a sparsity constraint to limit the number of genes used. While feature selection methods aim to eliminate redundant features (Hastie *et al.* 2000, Dettling and Bühlmann 2002, Yu and Liu 2004), neural networks leverage this redundancy in order to enhance the robustness of predictions with dropout techniques (Srivastava *et al.* 2014) or regularization linked to a graph representing interactions between genes (Ramirez *et al.* 2020). A general observation with statistical feature selection methods and classical ML models is that small subsets of genes are often sufficient to discriminate between phenotypes (Xiong *et al.* 2001, Krishnapuram *et al.* 2004, Li *et al.* 2017); this supports the existence of specific biomarkers. However, gene redundancy and the instability of selection procedures imply that the biological understanding derived from such specific gene subsets is far from complete (Xiong *et al.* 2001, Michiels *et al.* 2005, Venet *et al.* 2011, Waldron *et al.* 2014, Li *et al.* 2017).

In this work, we study whether neural networks and explainability techniques, shed new light on this longstanding issue of extracting comprehensive biological insights from gene expression levels. We evaluate the classification performance of the classifiers and the biological relevance of the top-ranked genes explaining their decisions. As far as we know, this topic hasn’t been explored in the literature.

Several classifiers, including shallow neural networks leveraging more complex relationships, graph-based neural networks exploiting gene interactions as well as LRs and XGBoost for comparison, are trained to differentiate cancer tissue samples. Genes rankings are derived from the neural networks using the integrated gradients explainability method (IG) (Sundararajan *et al.* 2017), e.g. as in Kimmel and Kelley (2021). Biological relevance is evaluated through over-representation analysis (ORA) (Khatri *et al.* 2012), which computes the overlap of the top-ranked genes with established gene sets representing various biological processes.

To elucidate the specific insights that neural networks can offer, the genes selected by integrated gradients are compared with those used by more interpretable ML models and those selected by standard feature selection methods and by statistical tests designed to identify differentially expressed genes [EdgeR (Robinson *et al.* 2010), DESeq2 (Love *et al.* 2014)]. Here, our objective is not to perform an exhaustive comparison analysis of feature selection methods, but rather to enhance the understanding of the neural networks used for phenotype classification.

Experiments are carried out on existing gene expression datasets from The Cancer Genome Atlas (TCGA; www.cancer.gov/tcga), the Therapeutically Applicable Research to Generate Effective Treatments programme (TARGET; www.cancer.gov/ccg/research/genome-sequencing/target), and the Genotype-Tissue Expression project (GTEx) (Lonsdale *et al.* 2013). The results indicate that the top-ranked genes identified by the neural networks vary, and differ significantly from those identified by statistical methods—presenting themselves significant variations.

Classification performance is maintained with small gene sets, which is coherent with existing literature on cancer classification from microarray (Xiong *et al.* 2001, Krishnapuram *et al.* 2004) and RNA sequencing data (Li *et al.* 2017). A strong redundancy of valuable information is also observed for neural networks, which is consistent with previous works on cancer classification (Xiong *et al.* 2001, Michiels *et al.* 2005, Venet *et al.* 2011, Waldron *et al.* 2014, Li *et al.* 2017). This redundancy contributes to the instability of the selected gene sets. Interestingly, in the simplest dataset (e.g. ttg-breast, see Table 1), the rankings derived from the explanations are intuitive: they are correlated with a t-statistic that measures the difference between the means of gene expression in two classes. We also observe that a classifier trained on genes selected by the statistical method DESeq2 or with a mutual information (MI) criteria outperforms a classifier trained on genes specifically selected for it. ORA reveals diverse biological processes, sometimes specific to a single method, suggesting different facets of explainability.

**Table 1.** Description of the various datasets and their associated classification tasks.

Dataset (source)	Task	# classes	# samples (min/max per class)	# genes
PanCan (legacy TCGA)	Tumour types	33	9853 (36/1095)	15 401
BRCA (GDC TCGA)	Healthy versus Tumour	2	1210 (113/1097)	13 946
BRCA-pam (legacy TCGA)	PAM50 classes	5	916 (67/421)	13 896
ttg-breast (TCGA TARGET GTEx)	Healthy versus Tumour	2	1384 (292/1092)	14 373
ttg-all (TCGA TARGET GTEx)	Healthy versus Tumour	2	17 600 (8130/9470)	14 368

In summary, we undertake a comprehensive analysis of gene expression profiling using emerging ML techniques by questioning classification efficiency and biological relevance. Comparisons with traditional statistical methods provide insights into the evolving landscape of molecular signature identification. The code and data are accessible at https://github.com/mbonto/XAI_in_genomics.

## 2 Methods

### 2.1 Setting

Let’s consider a gene expression dataset containing N training data samples of class c∈[[1;C]]. The class reflects the phenotypic state of a tissue. Here, it is a cancer type, a cancer subtype, or a healthy type. A data sample is a feature vector $x\in R^{G}$ containing the average expression of G genes within a tissue sample. The true class c of a sample is represented by a one-hot vector $y\in R^{C}$ having value 0 everywhere except for $y_{c}=1$. The Euclidean norm is written ‖·‖.

### 2.2 ML models for classification

To solve a classification task, a supervised model $f:R^{G}\mapsto R^{C}$ learns to map the features x of a data sample to a vector $\hat{y}\in R^{C}$ whose coefficients ${\hat{y}}_{c}$ represent the probability of belonging to class c. Four types of models are used in the present work: MLP, GNN, LR, and XGBoost for comparison.

#### 2.2. 1 Logistic regression

LR models with L2 or L1 regularization for both binary (C=2 classes) and multi-class (C>2) classification problems are considered. In the binary case, a sigmoid function is applied to compute class probabilities (Bishop 2006). Given the parameters $W\in R^{1\times G}$, b∈R, and σ(z)=1/(1+exp(−z)) the sigmoid function, the model is f(x)=σ(Wx+b). In the multi-class case, a softmax function is used instead of σ for the probabilities (Bishop 2006). Given the parameters $W\in R^{C\times G}$ and $b\in R^{C}$, $f(x)=\text{softmax}(\mathrm{Wx}+b)\;\text{with}\;\text{softmax}(z_{g})=\frac{\;\text{exp}(z_{g})}{\sum_{f}\;\text{exp}\;(z_{f})}.$ Two versions of LR, LR+L2, and LR+L1, are considered. LR+L2 is trained with a L2 penalty, a regularization term equal to the squared values of the parameters. LR+L1 uses a L1 penalty term equal to the absolute values of the parameters; it has the advantage of sparsity. As the number of non-zero parameters is minimized, the interpretability of potential candidate genes relevant for phenotypes is enhanced. These regularization techniques both prevent the model from over-fitting to the training data, and improve the generalization performance (Bishop 2006).

#### 2.2. 2 Multilayer perceptron

A MLP is a neural network containing a series of fully connected layers followed by a LR (Goodfellow *et al.* 2016). Formally, $G^{\left[l\right]}$ is the number of hidden features after layer l with $G^{\left[0\right]}=G$. In the binary case, $f(x)=\sigma (b^{\left[L\right]}+W^{\left[L\right]}\text{ReLU}(\ldots \text{ReLU}(b^{\left[1\right]}+W^{\left[1\right]}x)))$. The parameters learned within layer l are $W^{\left[l\right]}\in R^{G^{\left[l\right]}\times G^{\left[l-1\right]}}$ and $b^{\left[l\right]}\in R^{G^{\left[l\right]}}$. ReLU is the Rectified Linear Unit function. For the multi-class case, the sigmoid of the last layer is replaced by softmax. Here, the MLP architecture also includes a batch normalization function to each layer, which stabilizes training and improves generalization (Goodfellow *et al.* 2016). In the experiments, shallow MLP with one or two layers are used (see Section 2.5.3).

#### 2.2. 3 Graph neural network

A GNN (Zhou *et al.* 2020) incorporates gene pairs relationships using a graph structure. Following (Ramirez *et al.* 2020), a graph is created by connecting co-expressed genes through Pearson correlations. This generates a graph G, with nodes V={1,…,G} representing genes and edges E denoting relationships between gene pairs. The edge weights (i.e. thresholded correlations) are stored in the adjacency matrix $A\in R^{G\times G}$. The selection of the threshold is discussed in the Section 2.5.3. As in Ramirez *et al.* (2020), the GNN architecture includes one or two graph convolutional layers (Kipf and Welling 2017), each followed by a graph coarsening procedure [Graclus (Dhillon *et al.* 2007)], and a final LR.

Formally, F[l] is the number of features associated with a gene after layer l. Initially, the only feature is the gene expression value. The first graph convolutional layer is $\text{ReLU}(b^{\left[1\right]}+{\tilde{D}}^{-\frac{1}{2}}\tilde{A}{\tilde{D}}^{-\frac{1}{2}}xW^{\left[1\right]})$ with the data sample $x\in R^{G\times 1}$, $\tilde{A}=A+I$, and $\tilde{D}$ the degree matrix of $\tilde{A}$ (Kipf and Welling 2017). The same parameters $W^{\left[1\right]}\in R^{1\times F[1]}$ and $b\in R^{F[1]}$ are applied to all genes. The graph coarsening layer (Dhillon *et al.* 2007) combines pairs of adjacent nodes into a single node, preserving maximal features. New edges result from the union of previous edges, with associated weights summed.

#### 2.2. 4 XGBoost

XGBoost (Chen and Guestrin 2016) is a powerful ML algorithm based on decision trees. It uses an ensemble approach in which multiple trees are built sequentially, with each new tree correcting the errors of the previous ones. 

**Table 2.** Classification performance measured by balanced accuracy (%).

Dataset	XGBoost	LR+L1	LR+L2	MLP	GNN
PanCan	93.7	95.0	94.3	94.3 ± 0.3	92.1 ± 0.4
BRCA	99.7	99.7	98.5	99.5 ± 0.4	98.9 ± 0.6
BRCA-pam	92.2	92.3	90.7 ± 0.2	87.4 ± 1.8	87.1 ± 1.4
ttg-breast	98.3	99.7	99.2	99.4 ± 0.3	99.1 ± 0.1
ttg-all	99.4	99.5	99.5	99.6	99.4 ± 0.1

Standard deviations are computed from 10 replicates. They are not reported when below 0.05.

### 2.3 Gene selection and explainability for gene profiling

The objective is to identify biologically significant molecular signatures using gene expression data. Several methods assign a score $\phi_{g}$ to each gene g (its expression level being an input feature) indicating its relative importance for the sample phenotype class. These methods are categorized into three groups. (i) *Filter methods* rank genes based on a statistics measuring the amount of information they contain; filtering is done independently of any specific classifier. (ii) *Embedded methods* rank genes based on a score derived from a classifier; gene ranking is integrated into the training process of the classifier. (iii) *Post-hoc methods* rank genes based on a score computed after the training of a classifier; gene ranking occurs as a separate step after the classifier is trained.

#### 2.3. 1 Filter method—variance

Genes can be ranked in decreasing order of their variance (VAR) across samples in the dataset; filtering out genes with low VAR is often standard practice when preprocessing a dataset. By focusing on the gene expression data only and ignoring their corresponding phenotypes, this ranking method disregards true classes. Let $\bar{x}\in R^{G}$ be the average on all the training samples. Then, the score is:


(1) $$\phi_{g}^{\text{VAR}}=\frac{1}{N}\sum_{n=1}^{N}{\left(x_{g}^{n}-{\bar{x}}_{g}\right)}^{2}.$$

#### 2.3. 2 Filter method—principal component analysis

Genes can be ranked according to their contribution to the main directions of data variability. The higher the magnitude of a gene’s coefficient in the principal components, the more significant it is (Jolliffe 2002). Principal component analysis (PCA) focuses on the variability of the dataset without considering the classes. As large proportion of this variability is caught by the first principal component $v^{1}$, i.e. associated with the highest eigenvalue of the covariance $\Sigma \in R^{G\times G}$, only this one is kept here:


(2) $$\phi_{g}^{\text{PCA}}=|v_{g}^{1}|.$$

#### 2.3. 3 Filter method—MI

To account for higher order terms in probabilities, genes are ranked in decreasing order of the MI $I(X_{g};Y)$ shared between the expression $X_{g}$ of a gene g, and the classes represented by a discrete variable Y (Ross 2014). Then:


(3) $$\phi_{g}^{\text{MI}}=I(X_{g};Y).$$

#### 2.3. 4 Filter method—differential expression (EdgeR, DESeq2)

Popular bioinformatics tools are used to identify differentially expressed genes between experimental conditions (Costa-Silva *et al.* 2017). Once the expression distribution of a gene is modelled, a test for differential expression is performed and yields a *P*-value adjusted to account for multiple testing.

In this study, we consider EdgeR (Robinson *et al.* 2010) and DESeq2 (Love *et al.* 2014), two methods of this category that rely on different testing procedures. Genes are ranked according to their adjusted *P*-values; low adjusted *P*-values indicating a high statistical significance for differential expression. When more than two classes are considered, a test is performed for each possible pair of classes. The genes are ranked based on the minimal *P*-value obtained across all these tests. This ranking strategy reflects the presence of a significant difference for at least one pair of classes. With FiltMeth representing either EdgeR or DESeq2, the scores are:


(4) $$\phi_{g}^{\text{FiltMeth}}=-{\text{log}\;}_{10}(\text{adjusted}\;P{-\text{value}}_{g}^{\text{FiltMeth}}).$$

#### 2.3. 5 Embedded method—gains in XGBoost

In the context of decision trees, gain (Chen and Guestrin 2016) represents the reduction in error made when a feature is used to partition the data at a node. In other words, it measures the effectiveness of the feature in separating the data into more homogeneous classes. A high gain means that the chosen feature divides the data well, resulting in a better prediction. In algorithms such as XGBoost, gain is used to select features and optimize tree construction by choosing the most efficient splits.

#### 2.3. 6 Embedded method—magnitude of the LR weights

In a binary classification task using LR, the magnitude of a weight $W_{g}$ indicates the impact of gene g on the classification (Molnar 2022). Since we standardize gene expression distributions, the larger a weight magnitude the greater the influence of the corresponding gene on the classifier’s decision. Thus, the weight magnitudes can be used for ranking genes:


(5) $$\phi_{g}^{\text{LR}\;(\text{weight})}=|W_{g}|.$$

For multi-class, several linear functions (one for each class) are simultaneously learned. By analogy, genes are ranked based on the average of the absolute values of the parameters associated with a gene, hence:


(6) $$\phi_{g}^{\text{LR}\;(\text{weight})}=\frac{1}{C}\sum_{c=1}^{C}|W_{cg}|.$$

#### 2.3. 7 *Post-hoc* method—integrated gradients

Neural networks are not directly interpretable because of their functional complexity (Molnar 2022). To highlight the individual features that impact the most the decision of a neural network on a particular example, several explainability methods have been developed (Lundberg and Lee 2017). Here, the integrated gradients method (IG) (Sundararajan *et al.* 2017) is chosen to explain the decisions made by various gradient-based ML models, specifically LR, MLP, and GNN. IG is a gradient-based technique that is used a lot in the literature, in particular for its computational efficiency.

IG assigns a score function $\phi_{g}^{\text{local}}(x)$ to each gene g in each sample x to represent its importance for the model’s decision. The computation of the scores $\phi_{g}^{\text{local}}(x)$ contrasts the prediction for a sample x with a reference sample $x^{\prime }$ (baseline) according to:

$$\phi_{g}^{\text{local}}(x)=(x_{g}-x_{g}^{\prime })\int_{\alpha =0}^{1}\frac{\partial f_{c}(z)}{\partial x_{g}}{|}_{z=x^{\prime }+\alpha (x-x^{\prime })}d\alpha .$$

Here, $f_{c}(z)$ is the output of the model for class c at input z. To aggregate the decisions of a model, a global score $\phi_{g}^{\text{class}}$ for the importance of a gene g within a class c is derived using training samples that are correctly classified. If $N_{\text{class}}$ is the number of data samples of class c used to train a model, then:


(7) $$\phi_{g}^{\text{class}}=\frac{1}{N_{\text{class}}}\sum_{n=1}^{N_{\text{class}}}\frac{\left|\phi_{g}^{\text{local}}(x^{n})\right|}{\left\|\left[\phi_{1}^{\text{local}}(x^{n}),\ldots ,\phi_{G}^{\text{local}}(x^{n})\right]\right\|}.$$

The normalization ensures comparability across different samples. When multiple classes are studied, a global metric is derived by averaging the importance scores:


(8) $$\phi_{g}^{\text{IG}}=\frac{1}{C}\sum_{c}\phi_{g}^{\text{class}}.$$

The studied classes and the baselines used for each classification task considered in this article are detailed in the Section 2.5.4.

#### 2.3. 8 Interpreting neural networks with prediction gaps

Studying the impact of progressive gene masking on a model’s predictions provides a better understanding of the IG gene ranking. This masking, referred to as *experiment* 0 in the following, is performed by replacing the gene expression values in an original sample x truly belonging to class c, with the corresponding values from a reference sample $x^{\prime }$. For a sample ${\tilde{x}}_{m}$ with m masked variables, the prediction gap (PG) (Agarwal *et al.* 2022) is:


$$\text{PG}=\frac{1}{G}\sum_{m=1}^{G}\frac{\max(f_{c}(x)-f_{c}({\tilde{x}}_{m}),0)}{f_{c}(x)}\;.$$

**Figure 1.**
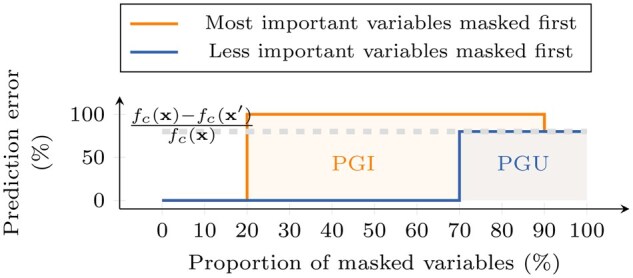
Illustration of PGI and PGU: prediction gaps on important (PGI) and unimportant (PGU) features, for an example x in a class c.

Genes can be masked using different orders on the predefined rankings ϕ: in descending order of importance, the metric is called PGI (I for important masked first); in ascending order of importance, it is called PGU (U for unimportant masked first). Figure 1 shows a sketch of what to expect of these metrics. Note that the transition between the original prediction of the data sample and the prediction of the baseline can be less smooth. If the rankings are good, PGU estimates the fraction of irrelevant features, while PGI calculates the fraction of important features that are not necessary for the models.

### 2.4 Interpretation of the selected genes

**Figure 2.**
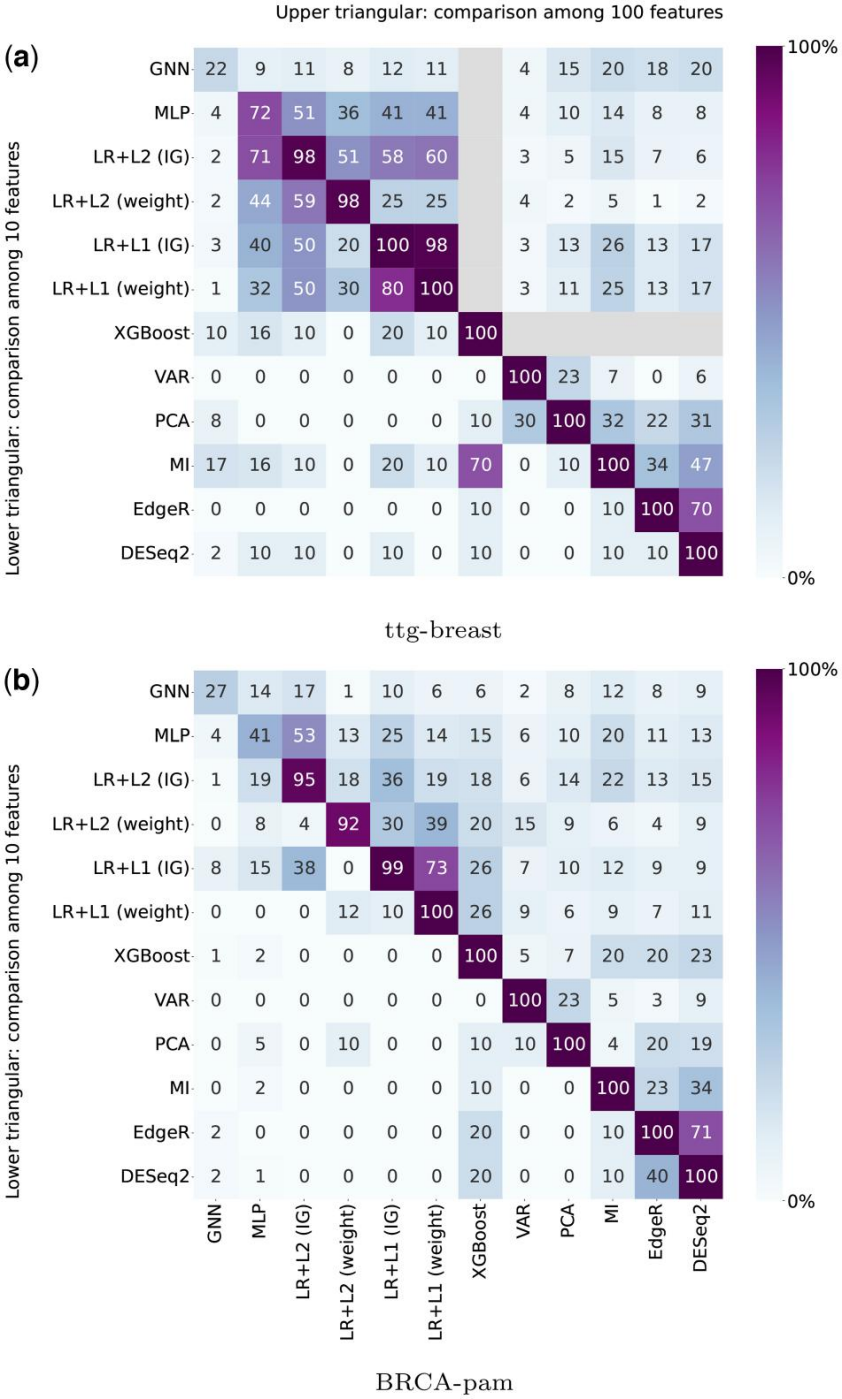
Heatmaps showing the percentage of common genes among the top 10 (lower) and top 100 (upper + diagonal) genes selected by each method. For ttg-breast, since XGBoost only uses 22 genes, the upper cells are masked. See the Subsection 2.4.1 for more details.

As the different selection methods generate distinct gene expression profiles, it is interesting to compare them. Rankings from classifier-based methods derive from learning, whose stability depends on the optimization process. For XGBoost, no variability is expected. In contrast, MLP and GNN may converge to different solutions, resulting in a significant variability. Both LR+L2 and LR+L1 theoretically converge to a unique set of parameters (see Boyd and Vandenberghe (2004) and Tibshirani (2013), respectively). However, practical factors such as the maximum number of iterations or rounding errors can introduce a very small variability. For instance, the percentage of common genes among the top 100 selected by these models over 10 replicates remains above than 92% across all datasets (Fig. 2 and Supplementary Fig. S1). To account for these variabilities, LR, MLP, and GNN models are trained 10 times with different initializations, which generates 10 ranking replicates. XGBoost, VAR, PCA, MI, EdgeR, and DESeq2 each generate a unique ranking per dataset.

#### 2.4. 1 Comparing the top-ranked genes across methods

For each dataset, heatmaps depicting the similarity of gene sets obtained by different methods are displayed (e.g. in Fig. 2). The lower/upper triangular part of the heatmap displays the percentages of common top 10/100 genes across different methods. When there are several ranking replicates, the percentages are averaged over all possible pairs. The diagonal of the heatmap shows the average percentage of common genes among the top 100 genes coming from ranking replicates of a method. For methods with a unique ranking, this percentage is 100%, by design.

#### 2.4. 2 Classification performance investigation

The analysis investigates the minimum number of genes needed for a model to achieve performance similar to using all genes. Two experiments are conducted. *Experiment* 1 aims at determining whether the $g_{H}$ highest-ranked genes contain enough information to discriminate the classes. Each model is trained using only the $g_{H}$ highest-ranked genes, with $g_{H}$ from 1 to 1000. *Experiment* 2 studies whether the $g_{L}$ lowest-ranked genes are informative. Each model is trained using the $g_{L}$ lowest-ranked genes, $g_{L}$ from 1 to 1000. For a model that produces several ranking replicates, the performance is averaged across all replicates.

#### 2.4. 3 Biological function investigation

After identifying the 100 most important genes for each method, an ORA (Khatri *et al.* 2012) is performed using gene sets from the Molecular Signatures Database Human collections (Liberzon *et al.* 2011; www.gsea-msigdb.org/gsea/msigdb/human/annotate.jsp). These gene sets, derived from the analysis of large repositories of gene expression data, encompass a variety of biological processes and pathological conditions. Specifically, the overlap of each gene list is calculated with the H hallmark gene sets (Liberzon *et al.* 2015), the C2 curated gene sets (including canonical pathways), the C4 computational gene sets (curated from cancer expression data), the C5 ontology gene sets, the C6 oncogenic signature gene sets, and the C7 immunologic signature gene sets. Results are filtered according to significance (false discovery rate q-value≤0.05). For each method, the top 10 over-represented gene sets are displayed. If any of these gene sets appears among the top 100 significant gene sets identified by another method, it is flagged. For ML-based methods, only one ranking is considered.

### 2.5 Materials for the experiments

#### 2.5. 1 Datasets

Bulk RNA-sequencing (RNA-seq) is a powerful technique for quantifying gene expression levels in tissue samples. The datasets analysed in this study originate from the TCGA Research Network, the TARGET initiative and the GTEx project (Lonsdale *et al.* 2013). They are accessible through the Genomic Data Commons (GDC) (Grossman *et al.* 2016; portal.gdc.cancer.gov) or the Xena browser (Goldman *et al.* 2020). TCGA (Tomczak *et al.* 2015) encompasses gene expression data from various human tumours and surrounding normal tissues, available in legacy and GDC versions. Notable distinctions between the two versions include the use of different human reference genomes (hg19 for legacy, hg38 for GDC) (Gao *et al.* 2019). The Pan-Cancer Atlas, derived from legacy TCGA, explores 33 tumour types (Hoadley *et al.* 2018). The TCGA TARGET GTEx dataset (TTG) integrates gene expression data from adult cancer tissues (TCGA), pediatric cancer tissues (TARGET), and non-diseased tissues (GTEx), all processed using a unified pipeline (Vivian *et al.* 2017).

This article focuses on classical classification problems, such as PanCan (Ramirez *et al.* 2020) or BRCA-pam (Rhee *et al.* 2018, Choi and Chae 2023). The datasets, detailed in Table 1, involve a limited number of samples for a large number of features (gene expression). Gene expression is measured by counting reads (fragment of RNA molecules) in a data sample. Preprocessing steps include the removal of genes with NaN values, those exhibiting a maximal expression level of 0 across the dataset, and low-expressed genes with fewer than five counts in over 75% of samples for each class. In the case of ttg-all, 27 samples are excluded due to null expression levels in more than 75% of the genes. Normalization involves scaling total counts in each sample to $10^{6}$, with subsequent ${\;\text{log}\;}_{2}$ transformation of the normalized counts. All datasets are available for download from our GitHub repository.

#### 2.5. 2 ML **m**odels training

The data is randomly split, with 60% of the samples used for training models and 40% for testing. Gene expression values are standardized using the means and standard deviations computed from the training set. XGBoost is trained with a learning rate of 1 using the XGBoost library (Chen and Guestrin 2016). With fixed hyperparameters, XGBoost’s training process is deterministic. For LR, MLP, and GNN, parameters of the models are learned through gradient descent, by minimizing the cross-entropy loss function (plus regularization). LR+L2 and LR+L1 are trained with the SAGA solver from Pedregosa *et al.* (2011) with a maximum of 1000 iterations. MLP is trained for 25 epochs using PyTorch’s SGD optimizer (Paszke *et al.* 2019) with a momentum of 0.9 and a weight decay of 0.0001. The initial learning rate is 0.1, decreasing to 0.01 after 13 epochs and to 0.001 after 23 epochs. GNN is trained for 15 epochs using PyTorch’s Adam optimizer (Kingma 2015) with a weight decay of 0.0001. The initial learning rate of 0.01 decreases to 0.001 after 8 epochs and to 0.0001 after 14 epochs. MLP and GNN can show a significant variability due to convergence to different local optima. In contrast, as LR+L2 and LR+L1 theoretically converge to a unique solution (see Boyd and Vandenberghe (2004) and Tibshirani (2013), respectively), no variability is expected. However, in one experiment, a small variability is observed (e.g. in Table 2, a standard deviation of 0.2% on the balanced accuracy for LR+L2 on BRCA-pam). To address this variability, LR, MLP, and GNN models are trained 10 times with distinct initialization seeds.

#### 2.5. 3 Hyperparameter selection

The hyperparameters of the models are selected with a grid search using a four-split cross-validation on the training data. The obtained parameters are in Supplementary Table S1. Note that for GNN, a threshold on the number of edges of each graph is set to control their density.

#### 2.5. 4 Methods for ranking genes

Gene rankings that need data samples to be calculated are generated using training samples only. All methods are implemented in Python except DESeq2 and EdgeR which are based on R packages. VAR, PCA, MI, and XGBoost are implemented using scikit-learn (Pedregosa *et al.* 2011). The code to include DESeq2 and EdgeR into a python script is inspired from Clarke *et al.* (2021). IG is implemented using the torch package called captum (Kokhlikyan *et al.* 2020). The IG scores are computed on the tumour class for BRCA, ttg-breast and ttg-all and on the tumour subtype classes for BRCA-pam. In these cases, the baseline $x^{\prime }$ with respect to which the scores are computed is the average of the normal training samples. The scores are computed on all classes for PanCan with respect to the average of the training samples.

## 3 Results

### 3.1 Gene expression data is informative on phenotypes

Five ML models, XGBoost, LR+L1, LR+L2, MLP, and GNN, are trained to classify gene expression data from tissues across several cancer types (PanCan), breast cancer and healthy surrounding tissues (BRCA), breast cancer and healthy tissues (ttg-breast), several cancer types and healthy tissues (ttg-all), and various subtypes of breast cancer (BRCA-pam) (Table 1). The classification performance is evaluated on data samples that have not been seen during training: 60% of the samples are used for training and the remaining 40% are for the evaluation. LRs, MLPs, and GNNs are trained 10 times with a different random initialization. XGBoost is deterministic. Table 2 reports the average balanced accuracy scores, correcting for class imbalance (average of recall obtained on each class). Unbalanced accuracies are similar, see Supplementary Table S2. The numbers of parameters for the models are in Table 3.

**Table 3.** Numbers of parameters learned by each model (averaged for non-zero parameters selected by LR+L1, and for GNN).

Dataset	XGBoost	LR (non-zero for L1)	MLP	GNN
PanCan	13 696	508 266 (13 575)	308 773	969 075
BRCA	75	13 947 (99)	279 001	26 187
BRCA-pam	740	69 485 (390)	278 085	118 484
ttg-breast	114	14 374 (206)	287 541	12 501
ttg-all	1746	14 369 (7376)	576 601	27 456

For XGBoost, the total number of parameters is the sum of the internal nodes and the leaves across all trees. For LR, *G* parameters are learned in the binary case, while G×C parameters are learned in the multi-class case, as implemented in scikit-learn (Pedregosa *et al.* 2011).

Across all datasets, phenotypes are predicted with balanced accuracy consistently exceeding 95%, except for BRCA-pam reaching 92%. High accuracies exceeding 99% are even achieved for datasets classifying cancer tissues against normal tissues. LR+L1 often outperforms other models (at the price of larger training time, see Supplementary Table S3), with XGBoost, LR+L2, and MLP following closely.

Good performance suggests that the models have learned informative patterns linking gene expression data to phenotypes. As LR+L1 performed similarly to XGBoost, MLPs, and GNNs, it suggests that precise classification can be achieved without necessarily learning interactions between genes; GNNs do not appear to benefit much from the graph of correlations.

### 3.2 Top-ranked genes obtained by explainability and statistical gene selection methods differ significantly

For each dataset, genes are ranked by the methods associated with the ML models (XGBoost, LR, MLP, GNN) or the statistical filtering methods (VAR, PCA, MI, EdgeR, DESeq2). For XGBoost, the calculated gains reveal that 22 genes are used for ttg-breast, 133 for BRCA-pam, 20 for BRCA, 528 for ttg-all, and 1568 for PanCan. Heatmaps are constructed to visualize the similarity of the top-ranked genes (Fig. 2 for datasets ttg-breast and BRCA-pam and Supplementary Fig. S1 for the other datasets).

For ttg-breast, LR exhibits more stable ranking replicates than MLP and GNN (diagonal of the heatmap). As expected, the more constant the classification performance (Table 2), the more similar the ranking between replicates. Across methods, the top-ranked genes may differ significantly. MLP and LR tend to select similar genes, possibly reflecting linear relationships with the class. LR with the embedded (weight) and *post-hoc* (IG) scores generate distinct rankings, especially for LR+L2. As IG is also applied to MLP and GNN, results across LR, MLP, and GNN are often more consistent when LR (IG) is considered instead of LR (weight). Thus, the next experiments are only conducted with LR (IG). For the other datasets, qualitatively similar results are observed, with even greater differences between the selected gene sets obtained by ML models and statistical methods.

### 3.3 Explaining the predictions with a small set of genes

We investigate the minimum number of genes required for a model to achieve performance comparable to using all genes. With XGBoost and LR+L1, the optimization process promotes sparsity, leading to the use of a small number of crucial genes, as shown by the number of non-zero parameters for LR+L1 in Table 3 and by the small number of genes used by XGBoost (22 for ttg-breast, 133 for BRCA-pam, 20 for BRCA, 528 for ttg-all, and 1568 for PanCan). The precise number of selected genes depends heavily on the choice of the hyperparameters. Still, the overall strong performance of LR+L1 and XGBoost indicates the ability to classify phenotypes effectively in these datasets with a limited number of genes.

*Experiment* 0 interprets the gene rankings derived by IG from LR, MLP, and GNN. The number of genes used by a trained classifier to make a decision is estimated by progressively masking the lowest-ranked genes without re-training. *Experiment* 1 measures the performance of ML models when they are trained from scratch using only the $g_{H}$ top-ranked genes identified by IG and feature selection methods. The goal is to assess whether the top-ranked genes are sufficient for classification.

**Figure 3.**
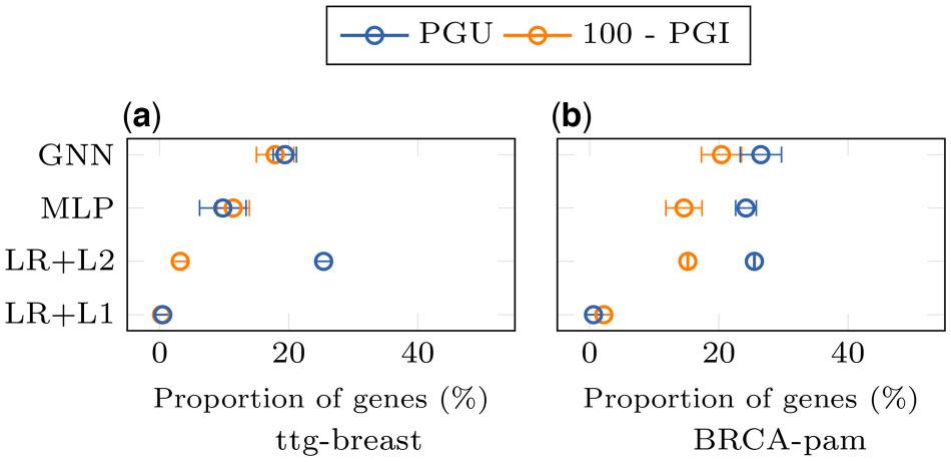
Impact of progressive gene masking on the predictions of ML models (*experiment* 0). Genes are masked by increasing (PGU) or decreasing order of importance (PGI) based on the rankings $\phi^{\text{IG}}$. For each data sample, PGU calculates the percentage of well-ranked genes that should remain unmasked to avoid disturbing a trained model. 100−PGI estimates the percentage of well-ranked genes that can be masked before disturbing the model. PGs are averaged over all training samples correctly classified. Error bars are standard deviations across replicates. See the Section 2.3.8 for more details.

For *experiment* 0, in Fig. 3, the Prediction Gap on Unimportant features (PGU) measures the minimum percentage of top-ranked genes that must remain unmasked to avoid disturbing the model’s decision. For ttg-breast, PGUs for LR+L1 is about 0.5% of genes (around 70 genes), which is less than the number of non-zero parameters in this model (Table 3). PGUs for MLP, GNN, and LR+L2 are 10%, 19%, and 25% of genes respectively (around 1400, 2700, and 3600 genes). For BRCA-pam, LR+L1 needs 0.6% of genes (around 80 genes), while other models have to keep a larger number of genes, between 24% and 27% (3300 to 3800 genes). Results on the other datasets are shown in Supplementary Fig. S2.

**Figure 4.**
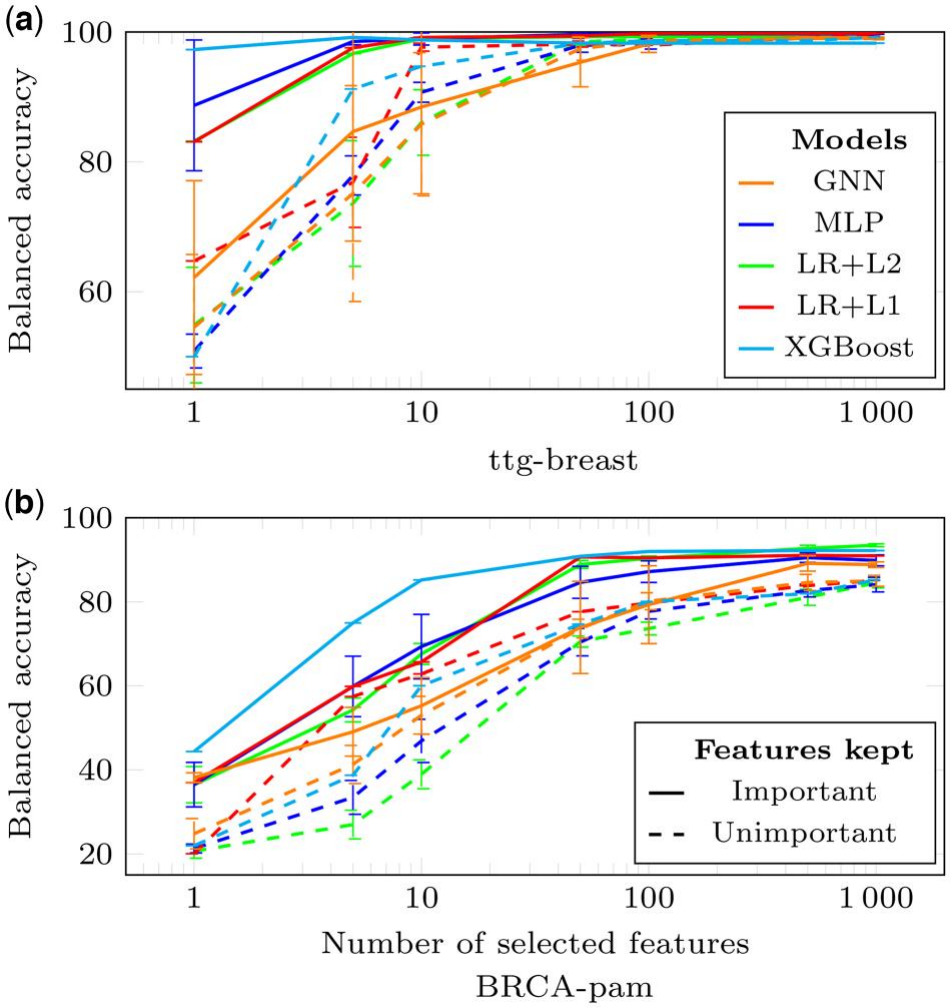
Classification performance shown for models trained on features deemed important (full lines, *experiment* 1) or unimportant (dashed lines, *experiment* 2) by the IG method for all models, except XGBoost that uses the gain metric. Balanced accuracies are presented relative to the number of features retained for ttg-breast (a) and BRCA-pam (b) datasets. Error bars are std from 10 replicates.

**Figure 5.**
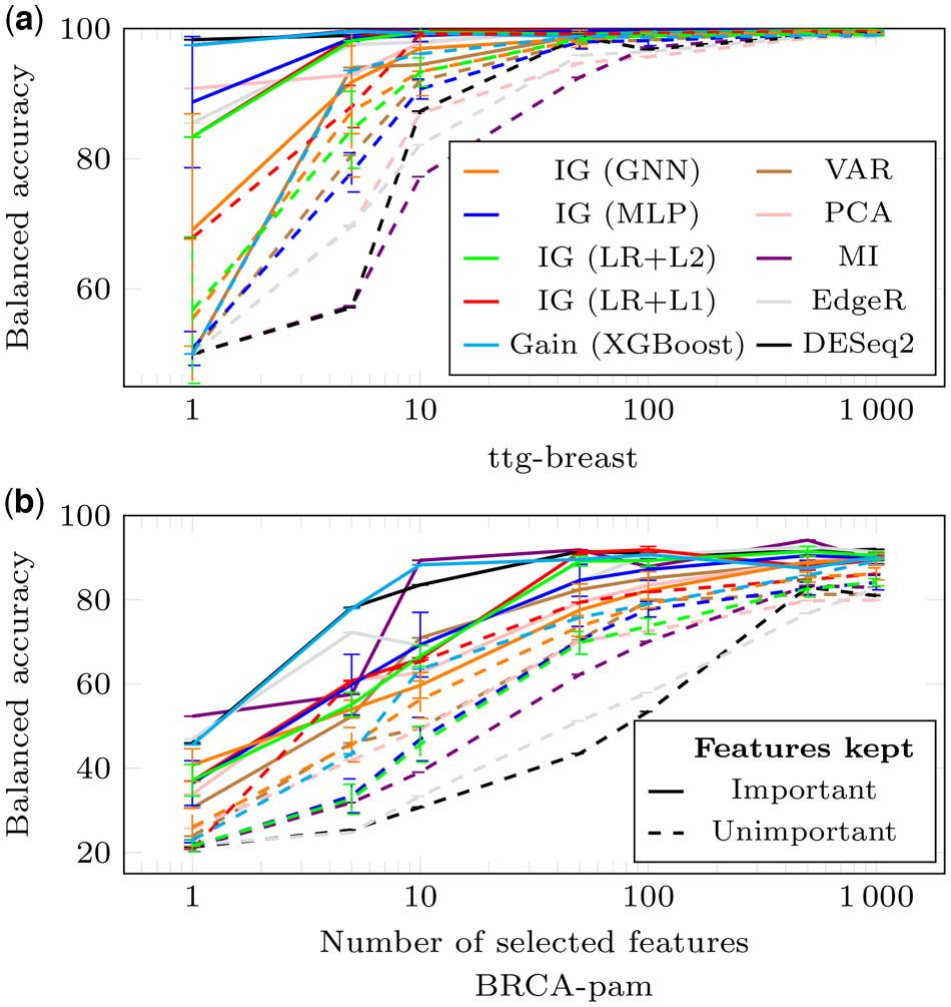
Classification performance of a MLP trained on features selected by various methods as indicated. The representation is coded as in Fig. 4.

For *experiment* 1, accuracies computed after re-training the models are in Fig. 4. For ttg-breast, the best 10 (resp. 100) genes are sufficient for MLP, LR+L1, LR+L2, and XGBOost (resp. GNN) to saturate the classification performance. For BRCA-pam, 500 genes are sufficient for all models. On both datasets, XGBoost, LR, and MLP obtain better performance than GNNs, which are disadvantaged because the graph structure is highly perturbed. Note that better classification performance can be obtained for MLP when trained on gene sets selected by other methods (Fig. 5). Results on the other datasets are in Supplementary Figs S3 and S4.

Thus, for a model trained on all genes, most genes except a very small set of the *best* genes can be masked without significantly perturbing predictions. Even smaller sets of top genes are sufficient to train models with high performance.

### 3.4 *Unimportant* genes matter

This analysis investigates whether the genes selected in the Section 3.3 exclusively contain relevant information. For that, we conduct *experiment* 0 by masking the highest-ranked genes without re-training the models and *experiment* 2 for which models are trained using the $g_{L}$ lowest-ranked genes.

For *experiment* 0, in Fig. 3, the Prediction Gap on Important features (PGI) measures the minimum percentage of worst-ranked genes that must remain unmasked to avoid disturbing the model’s decision. Here, the orange dots represent 100−PGI values, indicating the highest percentage of well-ranked genes that can be masked before perturbing the model. When the orange dots surpass the blue dots (PGU), it suggests that all the genes selected as important in the previous subsection can be masked without disturbing the model. Thus, the remaining information is sufficiently redundant. For ttg-breast, this occurs for MLP only. For LR+L2, masking a small proportion of the identified important genes is enough to disrupt the model. For BRCA-pam, LR+L1 can mask all the identified important elements. In contrast, for MLP, LR+L2, and GNN, masking a small proportion of the important genes is sufficient. These results show that when a large number of genes is perturbed, the model is disrupted. However, when the model is based on a small number of genes, these genes are not necessarily the only ones containing relevant information. Results on the other datasets are in Supplementary Fig. S2.

For *experiment* 2, with re-training, results are in Fig. 4 (dashed lines). Using the 100 lowest-ranked genes is sufficient to achieve optimal balanced accuracy on ttg-breast. For BRCA-pam, the gaps between the full lines (best-ranked genes) and the dashed lines (worst-ranked genes) are small. Results on the other datasets are in Supplementary Fig. S3. A similar experiment using randomly selected genes was conducted, although results are not shown in the figures for clarity. The resulting accuracies ranged between those achieved with the most and least important genes.

Similar classification performance can be achieved by keeping a relatively small set of lower-ranked genes, showing there is no unique set of informative genes. This dispersion is easily measurable for the binary classification tasks with a Welch’s unequal VAR *t*-test corrected for multiple testing. This statistical test quantifies the difference between the means of the distributions for each class, accounting for their average variances. Taking the ttg-breast dataset as an example, around 90% of the genes have an adjusted *P*-value below 5%. Thus, most genes carry enough information to distinguish the classes.

### 3.5 Stability across canonical gene sets

**Figure 6.**
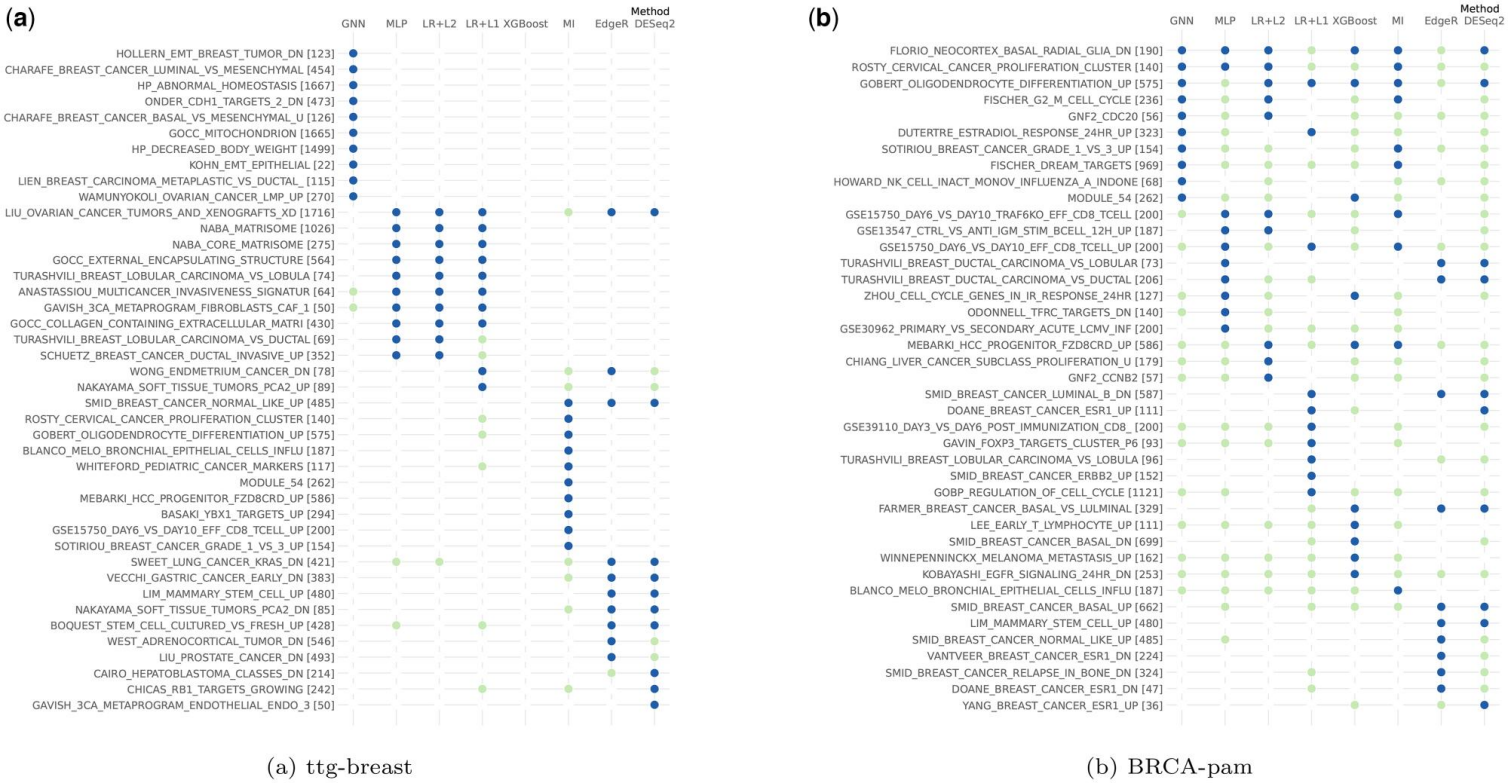
Canonical gene sets over-represented according to different methods. For each method, the 100 most important genes are used to identify the top 10 over-represented gene sets, represented by dark blue dots. Among these sets, those between the 10th and 100th positions in terms of over-representation are indicated with light green dots. For ttg-breast, since XGBoost uses only 22 genes, these 22 genes were used to identify over-represented gene sets; however, no such sets were found. See the Section 2.4.3 for more details.

The top-ranked genes identified by the different methods are compared with the content of gene sets known for their biological properties using an ORA. For each method, the top 10 over-represented gene sets are displayed in Fig. 6 (blue dots). When these gene sets appear among the top 100 significant gene sets identified by another method, they are flagged with a light green dot. See Supplementary Fig. S5 for the other datasets. Interestingly, different sets associated with (breast) cancer emerge across different methods. A general pattern of similarity emerges, with LR+L1, LR+L2, and MLP showing more consistent results, as well as notable agreement between EdgeR and DESeq2. However, the overlap between methods remains limited. The lack of overlap is particularly striking in the case of PanCan, and might be due to the complexity of the problem.

## 4 Discussion

Recently, many ML models have been proposed for classifying phenotypes from RNA sequencing data, with the goal of profiling genes discriminative for the phenotypes. These genes are identified through explainability techniques, which rank them by importance for a given model. Our objective is to evaluate the relevance of the explanations generated by ML models, examining whether they open new perspectives to elucidate complex biological pathways.

To address this question, experiments are carried out on human tissue samples coming from TCGA, TARGET, and GTEx databases. Two neural network architectures (MLP and GNN) are trained to classify a pathological state from other pathological or healthy states. Gene rankings are determined using the integrated gradient method (IG), a neural network-tailored explainability technique, and are compared with gene rankings derived from other ML models (LR, XGBoost) and also from classical feature selection methods such as MI and methods designed to identify differentially expressed genes (DESeq2, EdgeR). These models are representative of the main categories of methods put forward in the field of interpretable ML (LR, decision trees) and explainable ML (neural networks). Additionally, an ORA is conducted to explore the biological relevance of the top-ranked genes.

Several key insights emerge. First, the classifiers consistently achieve a balanced accuracy exceeding 95% in the majority of cases, with best performance being obtained by LR+L1. This result suggests limitations in leveraging complex relationships within the data. This is also supported by the fact that cross-validation experiments favoured shallow MLP with one or two layers and shallow trees for XGBoost. The classifiers can maintain the same performance using only a small set of top-ranked genes for training, which is coherent with existing literature (Xiong *et al.* 2001, Krishnapuram *et al.* 2004, Li *et al.* 2017). These genes are potential biomarkers for the studied pathologies. Yet, substantial differences appear in the genes identified across the different methods, highlighting the influence of inherent bias in each gene selection method.

However, good classification performance is also obtained with sets of lower-ranked genes, revealing both dispersion and redundancy of information in the gene space. Indeed, in trained neural networks and LRs, up to 20 % of the most important genes (100−PGI≃20%) can be masked without affecting classification performance. Similarly, models trained with lower-ranked genes achieve good performance. Comparable points can be made with randomly selected genes. These results are also in line with the literature (Xiong *et al.* 2001, Michiels *et al.* 2005, Venet *et al.* 2011, Waldron *et al.* 2014, Li *et al.* 2017). This likely contributes to the instability of gene selection. The redundancy of information in gene expression is not handled when ranking genes using explainability methods.

To gain insights into the genes preferentially used by ML models, looking at the correlation of rankings with a *t*-statistic computed from a Welch’s unequal VAR *t*-test is informative for binary classification problems. On ttg-breast dataset, scores from DESeq2, EdgeR, and MI are highly correlated with the *t*-statistic, with Spearman correlations around 0.9. LR and MLP exhibit lower correlation around 0.45. However, on a more complex dataset such as ttg-all, characterized by more heterogeneous data samples, correlations of the ML-based rankings with the *t*-statistic decrease. Surprisingly, GNN consistently exhibits low correlations, notably for ttg-all where the *t*-statistic distribution of the top 100 genes selected by IG is similar to those of genes chosen randomly. This highly questions the interpretability of genes by the GNN (IG) method.

In terms of classification performance, statistical methods looking for differentially expressed genes prove to be even better than ML methods in selecting a minimal set of informative genes (Fig. 5). A hypothesis is that during training, the MLP performance rapidly converges to 100% of accuracy, making the use of the most differentially expressed genes unnecessary.

The challenge is to determine which model is best suited for this medical data, particularly when the performance of the various models is comparable. It is relevant to prioritize the model offering the best interpretability (Rudin *et al.* 2022). However, the concept of interpretability in a biological context remains ambiguous. In this article, we address this concept by ranking genes according to their relative importance for the models. Our analyses reveal that while the top-ranked genes have a high discriminatory power, the lower-ranked genes also provide significant information. Nonetheless, from a biological perspective, this approach to interpretability does not yet offer a sufficiently clear perspective for precisely identifying the metabolic pathways underlying the observed phenomena.

Given the dispersed and redundant nature of relevant information, transitioning from individual genes to an exploration of cellular processes holds promise for enhancing our understanding of biological phenomena. Here, the ORA of the top 100 genes identified by the different methods highlights certain cellular processes, occasionally related to the studied pathologies. While some of them may be shared among methods, there is a substantial variability. The different genes identified by the methods may prove complementary, underscoring distinct processes leading to a phenotype. Further investigation of functional sets of genes, using for instance mechanistic experiments, could help to confirm this hypothesis.

ML methods able to automatically identify functional sets using biological knowledge, such as ontologies or metabolic pathways (Bourgeais *et al.* 2021), or able to determine a relevant scale of explanation through techniques like variable clustering, could provide a promising approach to unraveling complex biological processes.

In conclusion, this study provides valuable insights for researchers seeking biologically relevant molecular signatures of pathologies using ML. Investigating the impact of redundancy during the learning process and highlighting its influence for result interpretation are promising research directions. Additionally, the question of whether the reduced performance of GNNs stems from the graph representation or the learning process remains open (Brouard *et al.* 2024).

## Supplementary Material

vbae199_Supplementary_Data

## Data Availability

The gene expression data used in this article come from the TCGA, TARGET and GTEx databases. Detailed instructions for accessing these databases and recreating the datasets are provided at https://github.com/mbonto/XAI_in_genomics.
