# Supplementary material for: A comparative analysis of gene expression profiling by statistical and machine learning approaches
Source: Bioinform Adv. 2024 Dec 18;5(1):vbae199. doi: 10.1093/bioadv/vbae199 (PMC11783302; doi:10.1093/bioadv/vbae199)
Supplement: vbae199_Supplementary_Data [file vbae199_supplementary_data.pdf]

A Comparative Analysis of Gene Expression Profiling by Statistical and Machine Learning Approaches
Myriam Bontonou, Anaïs Haget, Maria Boulougouri, Benjamin Audit, Pierre Borgnat, Jean-Michel Arbona

Supplementary material

Datasets	XGBoost		LR		MLP		GNN		
	Estimator	Depth	λ (L1)	λ (L2)	Layer	Feature	Layer	Feature	k
PanCan	200	1	1	0.1	1	20	1	2	2
BRCA	25	1	0.1	0.01	1	20	1	2	2
BRCA-pam	50	1	0.1	1	1	20	1	2	10
ttg-breast	50	1	0.1	0.1	1	20	1	1	10
ttg-all	100	5	1	0.1	2	40	1	2	2

Table S1. Hyperparameters selected by grid search: the number of trees (chosen as 1, 5, 10, 25, 50, 100 or 200) and their depth (1, 3, 5 or 7) for XGBoost; the hyperparameter λ for the LR regularisation term (for trade-off with the cross-entropy loss) (0.01, 0.1, 1 or 10); the number of MLP layers (1 or 2), their number of hidden features (10, 20, 40 or 80); the number of GNN layers (1, 3 or 5), their number of hidden features (1 or 2), and k setting the number of graph edges to $k \times G$ (k is 2 or 10).

Dataset	XGBoost	LR+L1	LR+L2	MLP	GNN
PanCan	95.9	96.6	96.2	96.0 $\pm$ 0.2	94.5 $\pm$ 0.2
BRCA	99.4	99.4	97.3	99.3 $\pm$ 0.1	99.2 $\pm$ 0.1
BRCA-pam	91.5	91.8	89.5 $\pm$ 0.2	87.7 $\pm$ 1.3	87.5 $\pm$ 1.3
ttg-breast	99.1	99.5	98.7 $\pm$ 0.1	99.3 $\pm$ 0.2	99.2 $\pm$ 0.1
ttg-all	99.4	99.5	99.5	99.6	99.4 $\pm$ 0.1

Table S2. Classification performance measured by accuracy (%). Standard deviations are computed from 10 replicates; not reported when below 0.05.

Dataset	XGBoost	LR+L1	LR+L2	MLP	GNN
PanCan	751	58253	42564	74	404
BRCA	2	222	175	6	8
BRCA-pam	11	673	496	7	11
ttg-breast	4	262	207	10	12
ttg-all	49	6127	2703	136	150

Table S3. Average training duration (s) for each model.

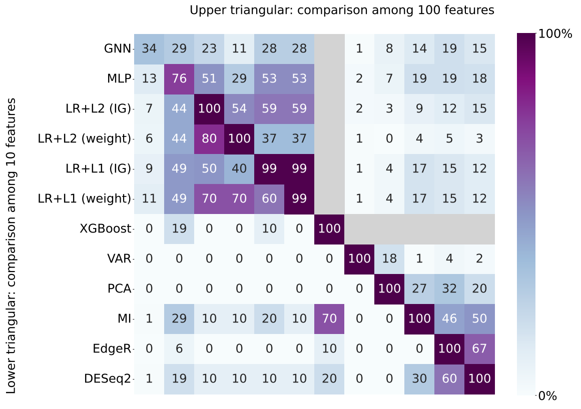

(a) BRCA

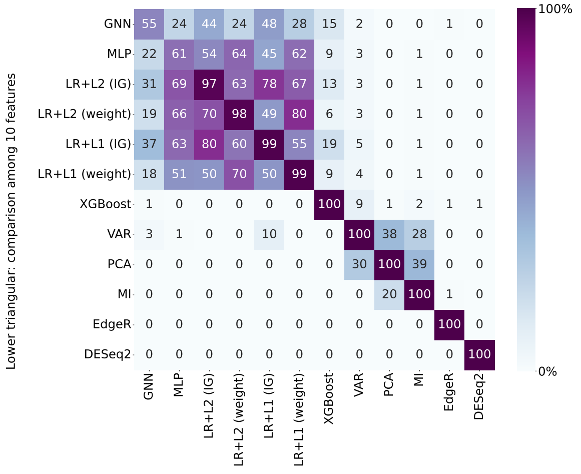

(b) PanCan

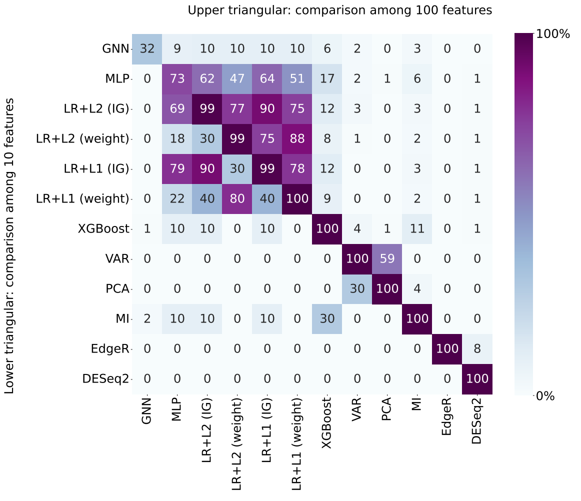

(c) ttg-all

Fig. S1. Heatmaps showing the percentage of common genes among the top 10 (lower) and top 100 (upper + diagonal) genes selected by each method. For BRCA, since XGBoost only uses 20 genes, the upper cells are masked.

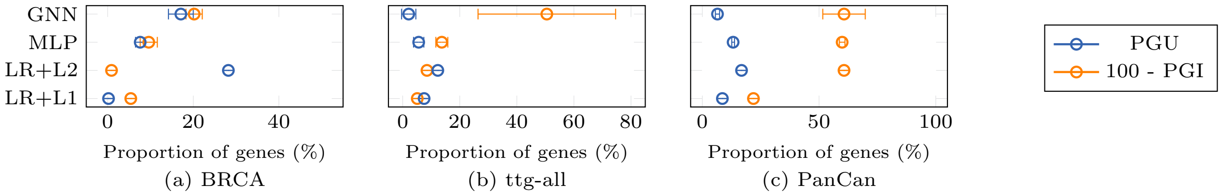

Fig. S2. Impact of progressive gene masking on the predictions of ML models (*experiment 0*). Genes are masked by increasing (PGU) or decreasing order of importance (PGI) based on the rankings ϕ^{IG}. PGs are averaged over all training samples correctly classified before masking, with error bars representing standard deviations.

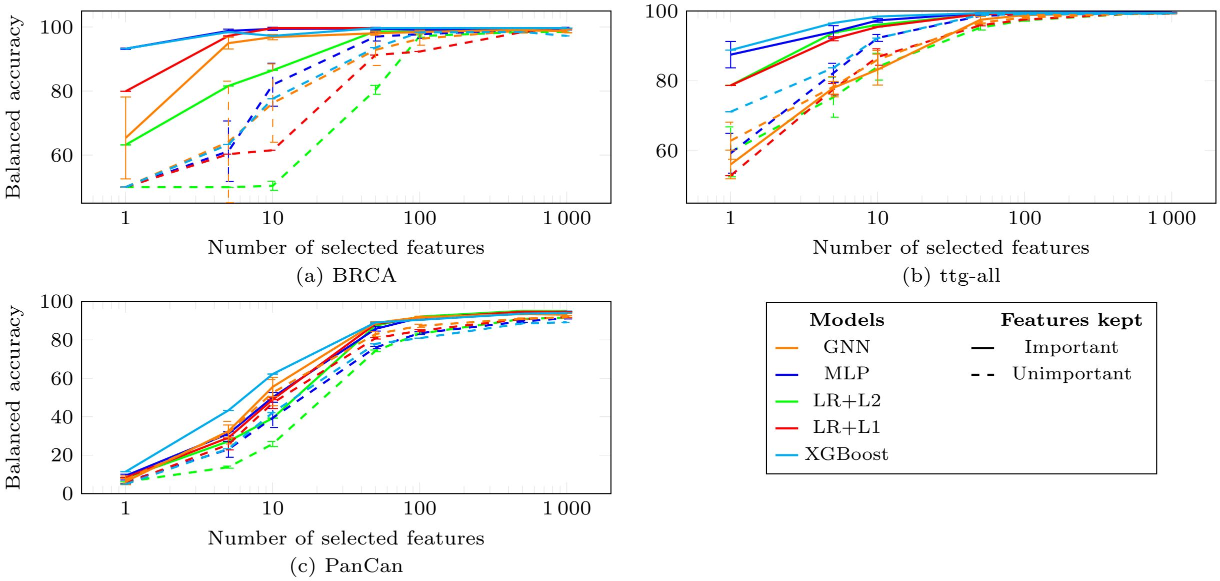

Fig. S3. Classification performance shown for models trained on features deemed important (full lines, *experiment 1*) or unimportant (dashed lines, *experiment 2*) by the IG method for all models, except XGBoost that uses the gain metric. Balanced accuracies are presented relative to the number of features retained using the specified models. Error bars are std from 10 replicates.

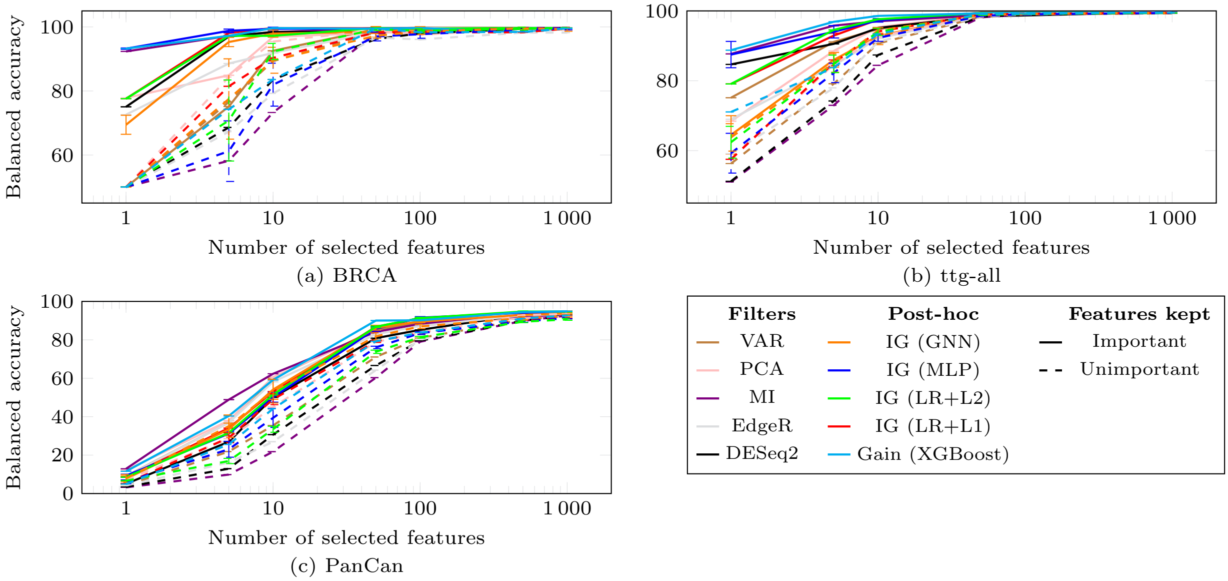

Fig. S4. Classification performance of a MLP trained on features selected by various methods as indicated. The representation is coded as in Fig. S3.

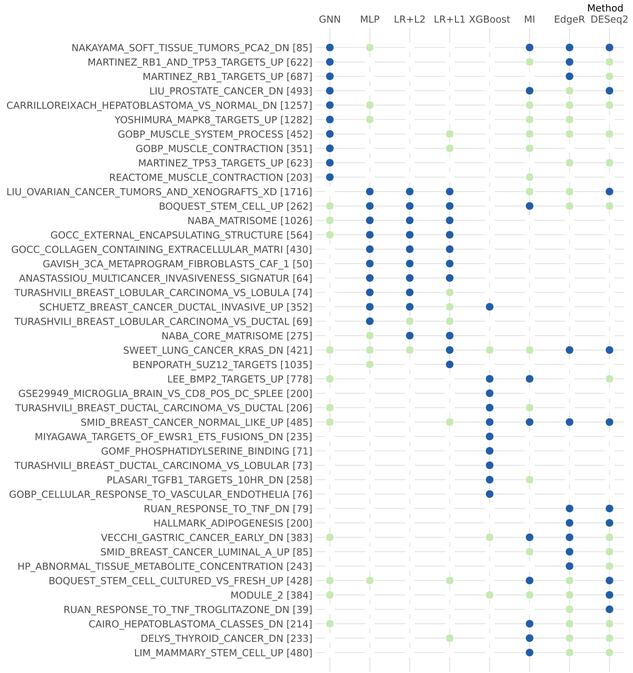

(a) BRCA

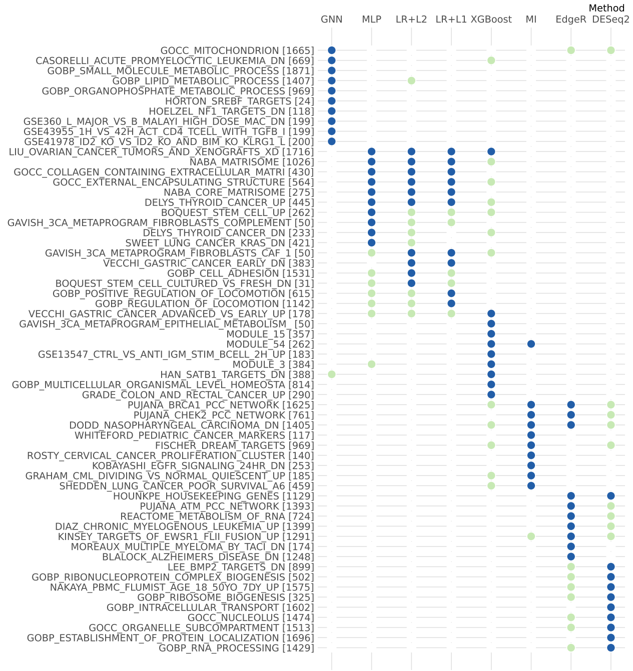

(b) ttg-all

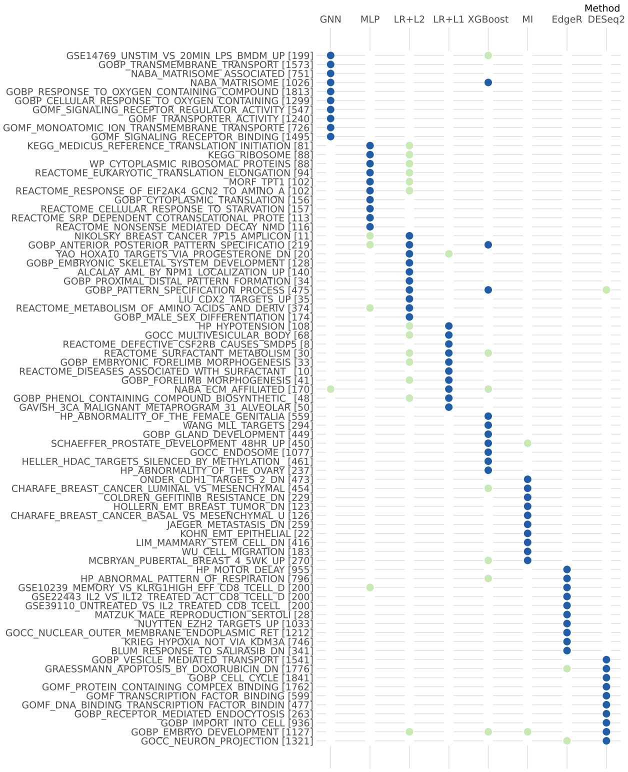

(c) PanCan

Fig. S5. Canonical gene sets over-represented according to different methods. For each method, the 100 most important genes are used to identify the top 10 over-represented gene sets, represented by blue dots. Among these sets, those between the 10th and 100th positions in terms of over-representation are indicated with light green dots. For BRCA, since XGBoost uses only 20 genes, these 20 genes were used to identify over-represented gene sets.
